# Supplementary material for: Integrating Artificial Intelligence for Advancing Multiple-Cancer Early Detection via Serum Biomarkers: A Narrative Review
Source: Cancers (Basel). 2024 Feb 21;16(5):862. doi: 10.3390/cancers16050862 (PMC10931531; doi:10.3390/cancers16050862)
Supplement: Supplementary file 1 [file cancers-16-00862-s001.zip › cancers-2828511-supplementary.docx]

Table S1: Summary of the diagnostic performance of the cfDNA biomarkers

| Biomarker | Cancer type | Sensitivity (%) | Specificity (%) | Reference |
| --- | --- | --- | --- | --- |
| Protein | | | | |
| OVA-1 | Ovarian cancer | 91 | 69 | (47) |
| ROMA: Human epididymis secretory protein 4 (HE4) and CA125 | Epithelial Ovarian cancer   - Pre-menopausal - Post -menopausal | 67.5  90.8 | 87.9  66.3 | (48) |
| 12 biomarkers:  Apolipoprotein A1 (ApoA1) and Apolipoprotein A2 (ApoA2); Cancer antigen 125 (CA125), Cancer antigen 19-9 (CA 19-9), alpha-fetoprotein (AFP), total prostatespecific antigen (tPSA), Carcinoembryonic antigen (CEA), Cytokeratinfragment 21-1 (Cyfra21.1), Human epididymis protein (HE4); beta-2 microglobulin (B2M); C-reactive protein (CRP), Transthyretin (TTR) | Stomach   - Stage 1,2 - Stage 3,4   Colon   - Stage 1,2 - Stage 3,4   Liver   - Stage 1,2 - Stage 3,4   Lung   - Stage 1,2 - Stage 3,4   Breast   - Stage 1,2 - Stage 3,4   Prostate   - Stage 1,2 - Stage 3,4 | 93.75,100  100, -  100, 90,  93.75, -  96.67,95.65  100,100  84.38,80.95  100,87.50  73.33, 78.57,  66.67,100  93.94,100  94.12,93.33 | 85.88  90.24  96.34  88.16  82.35  89.58 | (49) |
| cfDNA | | | | |
| MCBT 1 | Colorectum, esophagus, liver, lung, ovary, and pancreas  Stage 1-3 | 69.1 | 98.9 | (52) |
| MCBT 2 | Colorectum, esophagus, liver, lung, ovary, and pancreas | 75.1 | 95.1 | (52) |
| Therapy resistance mutations tumor derived cfDNA | **Colorectal**   - KRAS - RAS   **Metastatic Colorectal cancer**   - KRAS - RAS - BRAF   **NSLC**   - EGFR - EGFR – T790M - Pancreatobilliary carcinoma - Melanoma gastric carcinoma | 87.2  92.9  92  96.4  100  81.8  100  92.3  81 | 99.2  97.7  98  93  100  85.7  98.3  98.3  99 | (53) |
